# Serogroup A *Neisseria meningitidis* with Reduced Susceptibility to Ciprofloxacin

**DOI:** 10.3201/eid1410.080252

**Published:** 2008-10

**Authors:** Jacob Strahilevitz, Amos Adler, Gillian Smollan, Violeta Temper, Nathan Keller, Colin Block

**Affiliations:** Hadassah-Hebrew University Medical Center, Jerusalem, Israel (J. Strahilevitz, A. Adler, V. Temper, C. Block); The Chaim Sheba Medical Center, Tel Hashomer, Israel (G. Smollan, N. Keller)

**Keywords:** Neisseria meningitidis, meningococcal infections, ciprofloxacin, fluoroquinolones, drug resistance, epidemiology, molecular, letter

**To the Editor:** Reduced susceptibility to ciprofloxacin of *Neisseria meningitidis* has been reported with increasing frequency since 1992, mainly because of mutations in the quinolone resistance determining regions (QRDRs) of the gyrase and topoisomerase IV genes (*1*,*2*). Reduced fluoroquinolone susceptibility due to gyrase A mutations in serogroup A strains has previously been reported from a 2005 outbreak in Delhi, India (*1*). We describe 2 clinical isolates of serogroup A *N. meningitidis* with reduced ciprofloxacin susceptibility that were recognized in March 2003 and April 2006 in Israel, a country with low incidence of invasive meningococcal disease (<2/100,000/laboratory-confirmed cases/year) in which this serogroup accounts for <2% of cases (data from the National Center for Meningococci, Tel Hashomer, Israel).

The 2 isolates in question (M12/03 and M24/06; suffixes denote year of isolation) were compared with 2 fully susceptible strains, M44/01 and M23/00 (Appendix Table). MICs were measured by Etest (AB Biodisk, Solna, Sweden) on Mueller-Hinton agar (Difco Laboratories, Detroit, MI, USA) supplemented with 5% sheep blood. Demographic information was obtained from the Israel Ministry of Health Department of Epidemiology.

Chromosomal DNA was isolated by using the NucleoBond kit (Macherey-Nagel, Düren, Germany). The location of the QRDR in gyrase and topoisomerase IV genes was based upon prior studies in meningococci (Appendix Table) and on the complete sequence of strain *N. meningitidis* Z2491 (serogroup A; GenBank accession no. NC_003116). We amplified and sequenced extended regions encompassing the QRDRs by using the upstream and downstream primer pairs in *gyrA* (522 bases) 5′-GTTCCGCGTCAAAATATGCT-3′, 5′-CCGAAATTGACGGTTTCTTC-3′; *gyrB* (649 bases) 5′-GGTTTGACCTGCGTGTTGTC-3′, 5′-CGGCTGGGCGATATAGATG-3′; *parC* (635 bases) 5′-CACTATGGTTTGCCGTTTTG-3′, 5′-GATTTCGGACAACAGCAATTC-3′; and *parE* (610 bases) 5′-GGACAGGATGGCGATTTTG-3′, 5′-CGTCAGCAACTTCATCAACC-3′. PCR was performed by using *Taq* DNA polymerase (New England BioLabs, Beverly, MA, USA). DNA sequencing was performed using the ABI PRISM 3700 Genetic Analyzer (Applied Biosystems, Foster City, CA, USA). Screening for plasmid-mediated quinolone resistance genes was carried out by multiplex PCR amplification of *qnrA*, *qnrB*, and *qnrS* as previously described (*3*). Multilocus sequence typing (MLST) was carried out by using the primers, protocols, and databases available from the Neisseria MLST website (http://pubmlst.org/neisseria) (*4*).

The Appendix Table shows our results and condenses previously published findings. The ciprofloxacin MIC for M24/06 was 42- to 125-fold higher than for susceptible strains and consistently 2-fold higher than that for M12/03. We have not referred to our isolates as resistant, because M12/03 would be categorized as “intermediate” by Clinical Laboratory Standards Institute breakpoints (*5*). The extended QRDRs in *gyrA* and *parC* of M44/01 (susceptible) were identical to those of *N. meningitidis* Z2491. M24/06 and M12/03 had a Thr91Ile mutation in *gyrA*. M24/06 also had Asn103Asp, Ile111Val, and Val120Ile mutations in *gyrA* (Appendix Table; *1*). In M12/03, an Ala78Val mutation was found in *gyrA*, and new mutations Ile474Leu and Thr365Ala were found in *gyrB* and *parE*, respectively. No *parC* mutations were found.

Previous reports identified chromosomal mutations in *N. meningitidis* (Appendix Table). M24/06 and M12/03 possess the same Thr91Ile mutation in *gyrA* as a 2002 serogroup B isolate from Spain (Appendix Table) that had a similar increase in ciprofloxacin MIC (0.12 mg/L). The Thr91Ile mutation is homologous with the Ser83Leu mutation in *gyrA* of ***Escherichia coli*** that is responsible for a 60-fold increase in ciprofloxacin MICs (*6*). Further mutations in a primary target enzyme (gyrase) have been associated with additional 2-fold increases in the MIC of ciprofloxacin (*7*). The level of resistance observed in M24/06 might suggest additional mechanisms. An efflux pump mechanism is unlikely; we showed no reduction in MICs in the presence of reserpine (Appendix Table) and this organism was fully susceptible to penicillin, tetracycline, erythromycin, and Triton X-100 (data not shown). This finding suggests the absence of an efflux pump encoded by a mutated *mtrRCDE* (*8*). Neither M24/06 nor M12/03 had plasmid-mediated genes *qnr* genes or elevated kanamycin MICs, suggesting the presence of *aac(6′)-Ib-cr*. Both of these genes can confer low-level quinolone-resistance and facilitate the emergence of higher level resistance (*9*) (data not shown).

MLST showed that M24/06 and M12/03 did not derive from a single clone after selection of the T91I mutation. M12/03 was sequence type (ST) 2 and was isolated from a recent immigrant from Russia, which is the origin of most ST 2 strains deposited in the Neisseria MLST database (29/34 records; 85%). M24/06 was ST4789 in the ST5 clonal complex, isolated from a person who had immigrated many years previously from Romania. ST4789 has been encountered only once previously, in Dhaka, Bangladesh.

Disease associated with serogroup A *N. meningitidis* has been extremely unusual in Israel (*10*) and has remained rare. This serogroup comprised only 9 (1.9%) of all 463 isolates submitted during 1997–2006 (data from the National Center for Meningococci).

The isolates described in our study confirm that serogroup A should be added to the list of meningococci with the potential for reduced fluoroquinolone susceptibility and raise the question why they have appeared in a region with particularly low serogroup A meningococcal disease incidence while frequently encountered serogroups have remained fully susceptible. The importance of continuous monitoring for reduced ciprofloxacin susceptibility in these more prevalent serogroups has been emphasized by the recent replacement of rifampin by ciprofloxacin as the preferred agent for chemoprophylaxis of meningococcal disease in adults in Israel.

## Supplementary Material

Appendix Table Activity of ciprofloxacin against serogroup A Neisseria meningitidis with reduced and full susceptibility to fluoroquinolones and comparison of mechanisms associated with elevated MICs reported in N. meningitidis
