# Supplementary material for: Serogroup A Neisseria meningitidis with Reduced Susceptibility to Ciprofloxacin
Source: Emerg Infect Dis. 2008 Oct;14(10):1667–9. doi: 10.3201/eid1410.080252 (PMC2609879; doi:10.3201/eid1410.080252)
Supplement: Appendix Table — Activity of ciprofloxacin against serogroup A Neisseria meningitidis with reduced and full susceptibility to fluoroquinolones and comparison of mechanisms associated with elevated MICs reported in N. meningitidis [file 08-0252_appT-s1.pdf]

Publisher: CDC; Journal: Emerging Infectious Diseases
Article Type: Letter; Volume: 14; Issue: 10; Year: 2008; Article ID: 08-0252
DOI: 10.3201/eid1410.080252; TOC Head: Letter

Appendix Table. Activity of ciprofloxacin against serogroup A *Neisseria meningitidis* with reduced and full susceptibility to fluoroquinolones and comparison of mechanisms associated with elevated MICs reported in *N. meningitidis*

Location (isolates)	Serogroup	Source*	Ciprofloxacin MIC, mg/L	*gyrA* mutation	*parC* mutation	*gyrB* mutation	*parE* mutation
France 1996–98†	B	STD clinic	0.125	Asp-95→Gly	None		
Australia 1998‡	C	Blood, CSF	0.25	Asp-95→Asn	None		
Spain 2002§	B	CSF	0.12	Thr-91→Ile	None		
Argentina 2002¶	Y	CSF	0.12	None	None	None	None
Argentina 2002¶	B	Blood, CSF	0.06	Asp-95→Asn	None		
Spain 2007#**	Nongroupable	Sputum	0.25	Asp-95→Asn Asn-103→Asp Ile-111→Val Val-120→Ile	None		His-495→Asn
India 2005 (*1*)	A (12 outbreak isolates)			Thr-91→Ile Asn-103 → Asp Ile-111→Val Val-120→ Ile			
USA 2007–08 (*2*)	B (3 cases)	CSF	0.19	Thr-91→Ile			
Israel 2001 (M44/01)††	A	Blood	0.001	None	None	None	None
Israel 2000 (M23/00)	A	Blood	0.003	None	None		
Israel 2003 (M12/03)	A	Blood, CSF	0.064	Thr-91→Ile Ala-78→Val	None	Ile-474→Leu	Thr-365→Ala
Israel 2006 (M24/06)	A	Blood	0.125	Thr-91→Ile Asn-103→Asp Ile-111→Val Val-120→Ile	None	None	None

*STD, sexually transmitted disease; CSF, cerebrospinal fluid.
†Casin I, Gandry B, Lassau F, Janier M, Lagrange P, Collatz E. Decreased susceptibility to penicillin G (Pen), tetracyclines (Tet), and fluoroquinolones (Fq), and characterization of DNA gyrase mutations, in oropharyngeal and anogenital isolates of *Neisseria meningitidis* (Nme) in patients presenting at an STD clinic. In: Program and Abstracts of the 39th Interscience Conference on Antimicrobial Agents and Chemotherapy; San Francisco, CA; 1999 September 26-29; Abstract 2101. Washington: American Society for Microbiology; 1999.
‡Shultz TR, Tapsall JW, White PA, Newton PJ. An invasive isolate of *Neisseria meningitidis* showing decreased susceptibility to quinolones. Antimicrob Agents Chemother. 2000;44:1116.
§Alcala B, Salcedo C, de la Fuente L, Arreaza L, Uria MJ, Abad R, et al. *Neisseria meningitidis* showing decreased susceptibility to ciprofloxacin: first report in Spain. J Antimicrob Chemother. 2004;53:409.
¶Corso A, Faccone D, Miranda M, Rodriguez M, Regueira M, Carranza C, et al. Emergence of *Neisseria meningitidis* with decreased susceptibility to ciprofloxacin in Argentina. J Antimicrob Chemother. 2005;55:596–7.
#Enriquez R, Abad R, Salcedo C, Perez S, Vazquez JA. Fluoroquinolone resistance in *Neisseria meningitidis* in Spain. J Antimicrob Chemother. 2008;61:286–90.
**Nine additional strains reported; shown is a strain with mutations in *gyrA* similar to those found in isolate in present study.
††Identifiers of the National Center for Meningococci.
